# A Southeast Asian collaborative Delphi consensus on surveying risk factors for head and neck cancer screening and prevention

**Published:** 2022-09-13

**Authors:** DR Pan, E Juhlin, AN Tran, Q Wei, S Tang, AT Bui, NG Iyer, WT Lee

**Affiliations:** 1 Department of Head and Neck Surgery & Communication Sciences, Duke University, Durham, North Carolina, United States of America; 2 Department of Family Medicine and Community Health, Duke University, Durham, North Carolina, United States of America; 3 Department of Population Health Science, Duke University, Durham, North Carolina, United States of America; 4 Department of Medicine, Duke University, Durham, North Carolina, United States of America; 5 Duke Global Health Institute, Duke University, Durham, North Carolina, United States of America; 6 National Otolaryngology Hospital of Vietnam, Hanoi, Vietnam; 7 Department of Head and Neck Surgery, National Cancer Centre Singapore Duke-NUS Medical School, Singapore

**Keywords:** Head and neck cancer, screening, prevention, Delphi method, survey, Southeast Asia

## Abstract

The objective of this study was to determine high value questions for early detection and prevention of head and neck cancer by querying content experts on patient risk factors relevant to local communities in Southeast Asia (i.e., Vietnam, Laos, China, and Singapore). The Delphi method was employed using three rounds of asynchronous surveying which included participants among five different collaborating medical centers. 60 total survey items were assessed for consensus defined by a priori measures on the relative level of value of these questions for use in head and neck cancer screening. 77% of items reached a consensus and no items were concluded to be of low value despite differences in conclusions regarding relative importance. Survey items focused on patient demographic information and physical examination were examined across variables such as expert department affiliation, academic designation, and years of experience and found to be without statistically significant differences. However, with consensus items related to social risk factors, it was determined that participants who had 15 or more years of experience or identified as otolaryngologists rated these items at a relatively lower value than their peers with less experience (p < 0.0001, p = 0.0017) or outside the field of otolaryngology (p = 0.0101). This study explicitly identifies patient variables to consider in head and neck cancer screening that have not previously been comprehensively or methodically assessed in current literature. Increasing awareness of these risk factors may benefit the design and implementation of future head and neck cancer early detection and prevention programs in Southeast Asia and beyond as well as positively impact head and neck cancer outcomes.

## Introduction

Head and neck cancer is the sixth most common cancer worldwide and is especially damaging to patients due to its impact on basic human functions such as speaking, swallowing, and breathing [1]. The mortality and morbidity of head and neck cancers and its associated health and economic burden disproportionately impact low- and middle-income countries [2]. It has been estimated that 67% of head and neck cancer cases and 82% of deaths attributed to head and neck cancer occur in low- and middle-income countries [3]. Especially in low resource settings, head and neck cancers often present in advanced stages and result in poor outcomes. Challenges in low- and middle-income countries surrounding head and neck cancers include limited equipment and surgical capacity, lack of trained healthcare workers, and the paucity of organized head and neck cancer screening programs or national cancer control strategies [4,5].

One of the regions of the world that has the highest head and neck cancer incidence and mortality is Southeast Asia [6]. This is related to the presence of risk factors among the population such as betel nut, alcohol, and tobacco use [7–9]. Mortality bears large macroeconomic consequences; according to Patterson et al, the greatest cumulative loss in economic output due to head and neck cancer globally between 2018 and 2030 has been projected to be at $180 billion USD in the regions of Southeast Asia, East Asia, and Oceania [3]. The International Agency for Research on Cancer estimates that approximately 70% of new nasopharyngeal cancer cases globally are recorded in East and Southeast Asia [9]. Despite the large-scale impact of head and neck cancer in these regions, there is limited discussion in current literature regarding a comprehensive approach to systematically screen for and transfer knowledge about head and neck cancer in Southeast Asian communities.

The aim of this study was to determine high value screening questions for early detection of head and neck cancer in Southeast Asia by working alongside clinical content experts from partnering study site collaborators in Vietnam, Laos, China, and Singapore. Establishing an informed set of high value screening items is necessary to lay the foundation for the design and implementation of optimized head and neck cancer early detection and prevention programs in low- and middle-income countries in Southeast Asia.

## Materials and methods

### Delphi method and participants

**Figure 1:**
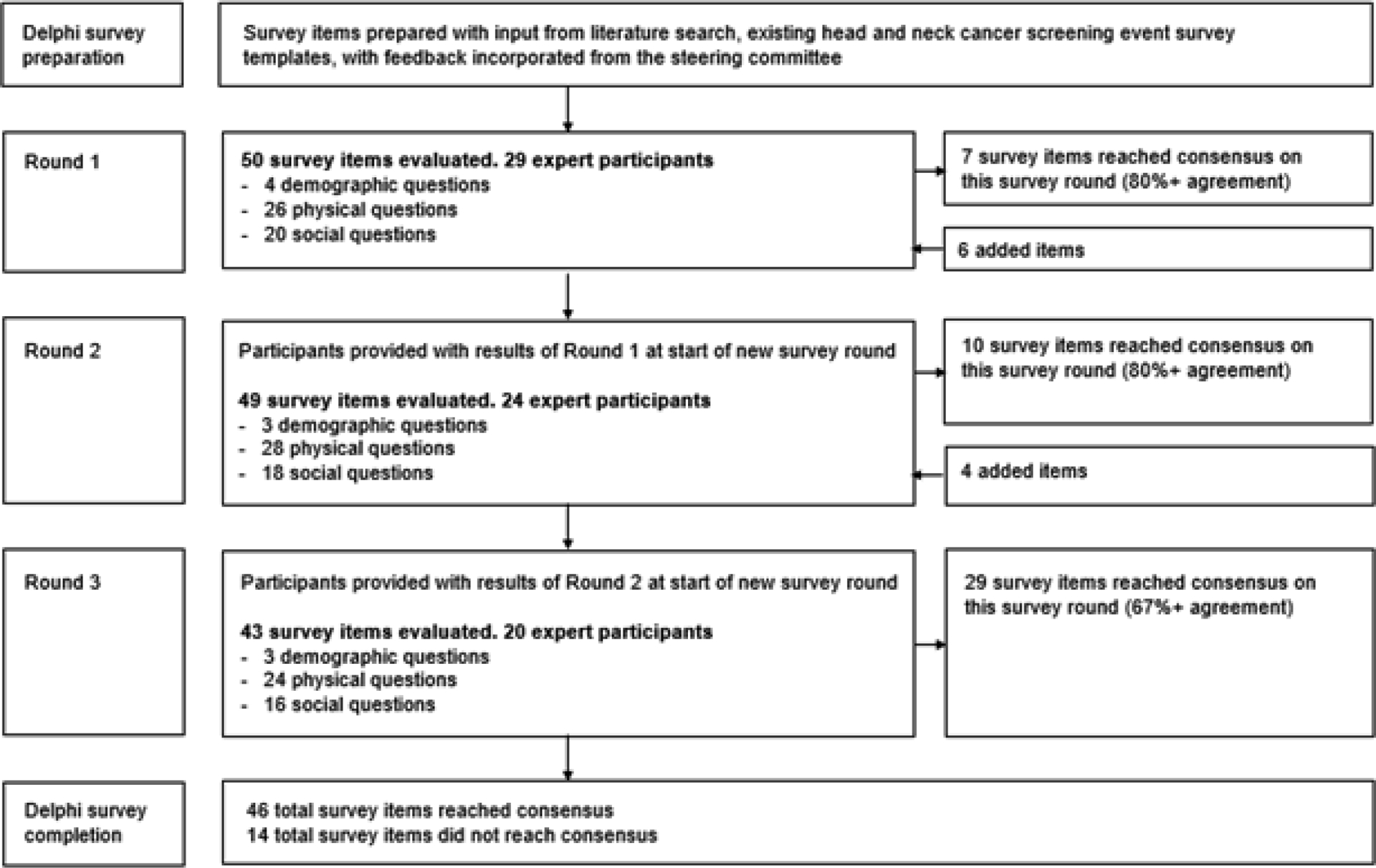
Overview of Delphi survey method with three round analyses.

An institutional review board exemption status from Duke University Medical Center was granted for this study (Protocol ID: Pro00109313). A head and neck cancer screening survey were developed to determine key screening questions for early detection which included items regarding head and neck risk factors, clinical history, and physical examination. These questions were prepared from literature review and expert steering committee input. A transdisciplinary team of stakeholders including local head and neck cancer and public health experts were invited to participate from the each of the participating study site collaborators: National Otolaryngology Hospital, Vietnam; Hue University of Medicine and Pharmacy Hospital, Vietnam; National Cancer Center at Mittaphab Hospital, Laos; Guangxi Medical University Cancer Hospital, China; and Singhealth Duke-NUS Academic Medical Center (National Cancer Centre Singapore and Singapore General Hospital), Singapore. The Delphi method was utilized to seek a consensus opinion through a multistage process in which experts completed a series of survey questions [10]. Three rounds of survey were conducted with feedback and results from the prior round distributed to all participants for subsequent rounds (Figure 1). Verbal consent was obtained from participants. Participants were also asked to provide personal demographic information including country, hospital, department affiliation, academic designation if applicable, and years of experience.

### Terminology and definitions

Three rounds of data collection were employed. Risk factors were identified as falling within three broad categories as they pertained to patients: demographic, physical (referring to clinical signs and symptoms endorsed or discovered on physical exam), and social (referring to aspects of social history). After each survey round, data were analyzed and the following predetermined criteria for agreement were applied. A threshold of 80% and above signified agreement. For each survey statement, the Delphi process stopped either when agreement was reached after which the statement would be removed from subsequent survey rounds or after completion of all three rounds. After the final round, a threshold of 67% and above was considered to have reached agreement.

Participants were asked to assess the utility of each survey statement by marking items as high value, moderate value, or low value. To minimize subjectivity of interpretation, a high value survey item was defined for participants as important to ask in most (>75%) patients. A moderate value survey item was defined for participants as important to ask in some (25–75%) patients. A low value survey item was defined for participants as important to ask in a minority (<25%) of patients. Participants could submit comments such as other items to review on subsequent rounds for each category of surveyed questions.

### Survey administration

A web-based Delphi method survey was employed from October 2021 to March 2022. Participants were emailed the survey and study data were collected and managed using REDCap (Research Electronic Data Capture) hosted at Duke University Hospital. REDCap is a secure, web-based software platform designed to support data capture for research studies, providing an interface for validated data capture, audit trails for tracking data manipulation and export procedures, automated export procedures for data downloads to common statistical packages, and procedures for data integration and interoperability with external sources [11]. Participants were given a unique link and allowed one entry per round. Data were exported from the REDCap database for descriptive analysis in Microsoft Excel (2022).

## Statistical analysis

Two-sample t-test statistical sub-analysis was performed via MedCalc Statistical Software version 20.027 (MedCalc Software bvba, Ostend, Belgium; https://www.medcalc.org; 2022). These comparisons were performed for several variables on consensus items by assigning a traditional three-point scale to survey questions: high value as 1, moderate value as 2, and low value as 3. These numerical assignments were not intended to hold mathematical significance other than to serve as a relative metric by which statistical differences in responses could be examined. Results are reported in the following section with statistical significance defined as p < 0.05.

## Results

The survey was distributed to 29 experts. A total of 20 out of 29 (69%) participants completed all three rounds; 29 out of 29 responded on Round 1, 25 out of 29 responded on Round 2, and 20 out of 25 responded on Round 3. Demographic data were reported on these experts who had fully participated, which can be found in Table 1. Most participants were from Vietnam. Otolaryngology was the most reported department affiliation followed by general surgery.

A total of 60 survey items were queried over three rounds. A consensus on value determination was achieved on 77% of questions surveyed: 12% of items reached a consensus on round one, 17% on round two, and 48% on round three (Figure 1). The questions that were determined to be of high value can be found in Table 2. The questions that were determined to be of moderate value can be found in Table 3. No survey items were agreed upon to be of low value. The remainder of questions that did not achieve a consensus by the final round are listed in Table 4.

Among the 46 survey items that achieved a consensus (Tables 2 and 3), sub-analysis was performed via two-sample t-test on several variables including department affiliation (Otolaryngology vs Non-otolaryngology), designation (Academic title vs Non-academic affiliation), and years of experience (<5 vs 5–14 vs 15+) to determine if there was any statistically meaningful difference in survey results based on these factors (Table 5). Sub-analysis did not reveal statistically significant differences between how demographic or physical items were scored across all examined variables. The presence of an academic title also did not reveal significant differences in how survey questions were valued. However, within the social item category, there were statistically significant differences appreciated in how these questions were valued when department affiliation and years of experience were considered. Otolaryngologists rated social items at a lower relative value compared to non-otolaryngologists (p = 0.0101). Participants with over 15 years of experience rated social items at a lower relative value compared to those with fewer than 15 years of experience (p < 0.0001, p = 0.0017).

## Discussion

Results of this survey indicate survey question consensus about the generalizability of various risk factors for head and neck cancer when applied in the context of screening. The majority (46 out of 60; 77%) of survey items examined reached a consensus by the conclusion of the Delphi process and of these, 32 out of 46 (70%) were determined to be of high value with 14 out of 46 (30%) deemed as moderate value. Per aforementioned methodology, high value reflected items that were important to ask in most (>75%) patients and moderate value reflected items that were important to ask in some (25–75%) patients. Given that there were no items that were agreed upon to be of low value, these collective survey items reflect the importance of factoring aspects of a patient’s demographic information, clinical physical examination findings, and social history into consideration for head and neck cancer screening. From the physical category of survey items, it appears that factors that influence function such as swallow, speech, or respiration were ranked higher in value than symptoms that could be considered as nonspecific complaints. The survey items that were ranked moderate value generally involved symptomatology with multiple possible etiologies or systemic concerns. Not surprisingly, social variables such as tobacco, alcohol, and betel nut quid use, or exposure were ranked as high value in accordance with their status as known risk factors for head and neck cancer in this region [12,13]. In fact, in a multicenter case-control study in East Asia, individuals with habits of using all three substances were at a significantly higher risk of head and neck cancer (OR = 20.6) compared to each substance alone (OR = 1.6, 2.3, 8.2, respectively) [13].

Interestingly, when consensus items were sub-analyzed, the only statistically significant difference was found with respect to the category of social items in which otolaryngologist rated these questions at a lower relative value compared to non-otolaryngologists. This same trend was also observed in expert participants with over 15 years of experience compared to those groups with fewer years. These findings can possibly be explained by the assumption that more experienced otolaryngologists more likely encounter an element of selection bias with the head and neck patients that are referred to them compared to their counterparts in other departments or with less years of experience. Such patients likely have already been initially evaluated at some point and present with persistent symptoms or functional deficits that signal for an evaluation by an otolaryngology specialist. By this encounter, it is possible that social history is a less pertinent factor as the diagnosis of potential head and neck cancer would rely on a thorough visual and tactile physical examination with biopsy of suspicious lesions as the next step.

This is the first study of expert consensus on key questions for head and neck cancer to be used for screening in Southeast Asia, influenced by inevitable epidemiological differences of head and neck cancer between the Western and Eastern hemispheres. The burden of head and neck cancer with a paucity of described organized head and neck cancer early detection and prevention programs in Southeast Asia prompts the necessity for delineating an intentional and informed approach to screening.

Several global studies have demonstrated significant, measurable success of screening programs with respect to patient education on prevention as well as detection of head and neck cancer [14–18]. According to perhaps the highest impact study in this field conducted in India and the only randomized control trial to examine the effect of screening of oral cancer on mortality, it has been concluded that screening has the potential to prevent at least 37,000 deaths from oral cancer worldwide every year [19]. Data from a Korean population-based study by Wee et al revealed that the risk of head and neck cancer and oropharyngeal cancer was significantly higher in individuals who participated in oral health screening programs compared to those who participated in routine health check-ups only [20]. A large-scale population-based screening program targeting cigarette smokers and betel quid chewers in Taiwan led to an estimated 21% reduction in stage III or IV oral cancer diagnoses and a 26% reduction in mortality when participants were followed for a median follow up of 4.5 years [12]. In these studies, patients were screened for oral habits, such as smoking and alcohol use, but descriptions of other screening items were not included. There was an emphasis on appropriately trained professionals to recognize clinical diagnoses such as oral leukoplakia, erythroplakia, submucous fibrosis, verrucous hyperplasia, or ulcers by visual inspection, but no mention of surveying patient reported symptomatology [12,19,20]. In addition, a recent study by Thampi et al. [21] has demonstrated the feasibility of training community healthcare workers in resource-constrained environments. Community healthcare workers in India who were trained by dentists to perform oral cancer screening over a one-day hands-on workshop achieved an overall screening sensitivity and specificity of 96.69% and 98.69%, respectively, when assessing a total of 1200 individuals during home visits [21].

In current literature, a popular screening form that has been employed by numerous studies, if mentioned, is a form sponsored by the Head and Neck Cancer Alliance’s Oral, Head and Neck Cancer Awareness Week which is a free screening initiative hosted at multiple sites across the United States [15,22–24]. This form assesses basic demographic information along with 25 statements for patient response. Our study hopes to expand on the quantity and quality of utilized head and neck cancer questionnaire items for a more comprehensive and population-targeted survey. Future survey protocols may consider including high value survey items and deciding to include items deemed by our study as moderate value or not reaching consensus based on the context specific needs of an examined patient community. While there could be perceivable benefit for the implementation of further head and neck screening experiences in Southeast Asia, these data may also guide future head and neck cancer research initiatives in other communities.

There are several limitations to this study. The Delphi survey method allows for the participation of experts in an iterative multistage process to convert opinion into group consensus [25]. In a systematic review of Delphi studies, the definition of consensus is varied but usually established a priori with 75% as the median threshold among examined studies [26]. However, this lack of standardization in approach can limit the reliability of the results. It is also important to note that a consensus does not imply that a definitive or correct answer has been established as consensus can change based on the number of participants and should be used primarily for structuring discussion about relative importance of a topic. While our study’s participant number of 20 exceeds the median sample size of 17 reported from previous studies, there are inherent subjective limitations to the methodology of the Delphi process as mentioned that should be considered while interpreting results [10]. Additionally, although sub-analyses were conducted with variables related to the expert participant demographics, sub-analyzing by country to determine if there were differences in consensus specific to varying local communities was not performed due to limited sample sizes from participants of some countries. While efforts were intentionally made in this study to include a heterogenous panel of stakeholders, it is also important to maintain a broad perspective on provider input as head and neck cancer often involves those outside of the fields of surgery or oncology.

For effective screening, the diagnosis of head and neck cancer in its early stages depends on prompt recognition of risk factors, signs, and symptoms by the patient or referring providers such as primary care physicians, dentists, or speech language pathologists. Further research efforts will focus on incorporating these findings into the design and implementation of early detection and prevention programs in the low- and middle-income communities of Southeast Asia.

## Figures and Tables

**Table 1:** Expert participant demographics (n = 20).

Countries	Number (%)
Vietnam	11 (55)
China	3 (15)
Singapore	3 (15)
Laos	3 (15)
	
**Department affiliation**	
Otolaryngology	13 (65)
Medical Oncology	2 (10)
Oncology Pharmacology	1 (5)
Oral Surgery	1 (5)
General Surgery	3 (15)
	
**Designation**	
Professor	2 (10)
Associate Professor	4 (20)
Assistant Professor	1 (5)
Physician/Surgeon	13 (65)
	
**Years of experience**	
<5	6 (30)
5–14	8 (40)
15+	6 (30)

**Table 2:** List of high value survey questions.

Demographic	Physical	Social
Age >40 years	Neck mass(es)	Current smoker status
	Dysphagia	Time frame of smoking
	Hoarseness	Packs per day of smoking
	Difficulty breathing	Prior smoker status
	Unilateral nasal obstruction	Prior pack-year smoking history
	Head and neck pain	Quit date of smoking
	Odynophagia	Exposure to second-hand smoke
	Stridor	Current regular alcohol use
	Voice change	Amount of alcohol use
	Oral or nasal bleeding	Time frame of alcohol use
	Oral ulcers	Prior alcohol use
	Oral leukoplakia	Prior amount of alcohol use
	Oral erythroplakia	Quit date of regular alcohol use
		Betel nut quid use
		Personal head and neck cancer history
		Family head and neck cancer history
		Personal other cancer history
		History of prior radiation exposure

**Table 3:** List of moderate value survey questions.

Demographic	Physical	Social
Education level	Otalgia	
	Poor dentition	
	Globus sensation	
	Change in sense of smell	
	Foul taste	
	Halitosis	
	Toothache	
	Unintentional weight loss	
	Fever	
	Cough	
	Ill-fitting dentures	
	Dizziness or vestibular dysfunction	
	Night sweats	

Moderate value survey item: deemed important to ask in some (25–75%) patients.

**Table 4:** List of survey questions which did not achieve a consensus.

Demographic	Physical	Social
Sex	Bilateral nasal obstruction	Awareness of head and neck cancer
Occupation	Hearing loss	History of tuberculosis
Living conditions	Headache	Use of smokeless tobacco
		Habit of eating spicy or hot foods
		Number of sexual partners
		Same-sex partners
		History of sexually transmitted disease
		Participation in oral sex

**Table 5:** Variable sub-analysis performed via two-sample t-test on consensus items.

Variables	Difference* (95% confidence interval)	p-value
**Department affiliation:** Otolaryngology vs Nonotolaryngology		
Demographic	0.137 (−0.2582 to 0.5329)	Non-significant
Physical	−0.084 (−0.1941 to 0.0262)	Non-significant
Social	−0.126 (−0.2213 to −0.0302)	0.0101
		
**Designation:** Academic title vs Non-academic affiliation		
Demographic	−0.208 (−0.5899 to 0.1732)	Non-significant
Physical	−0.034 (−0.1412 to 0.0736)	Non-significant
Social	0.008 (−0.0857 to 0.1022)	Non-significant
		
**Years of experience:** <5 vs 5–14 vs 15+		
Demographic (<5 vs 5–14)	0.271 (−0.2245 to 0.7662)	Non-significant
Demographic (5–14 vs 15+)	0.063 (−0.3643 to 0.4893)	Non-significant
Demographic (<5 vs 15+)	0.333 (−0.1499 to 0.8166)	Non-significant
Physical (<5 vs 5–14)	0.000 (−0.1316 to 0.1316)	Non-significant
Physical (5–14 vs 15+)	0.103 (−0.0216 to 0.2270)	Non-significant
Physical (<5 vs 15+)	0.103 (−0.0306 to 0.2360)	Non-significant
Social (<5 vs 5–14)	−0.071 (−0.1634 to 0.0223)	Non-significant
Social (5–14 vs 15+)	0.286 (0.1830 to 0.3895)	<0.0001
Social (<5 vs 15+)	0.216 (0.0820 to 0.3494)	0.0017

* Difference calculated by correlating traditional three-point scale with survey responses: high value as 1, moderate value as 2, and low value as 3.
